# Prostate Cancer Diagnosis by Transurethral Resection of the Prostate Is Associated with Compromised Oncologic Outcomes Post-Prostatectomy

**DOI:** 10.3390/cancers18040569

**Published:** 2026-02-09

**Authors:** Abdullah Al-Khanaty, Marlon Perera, Brendan Yanada, Melanie Evans, Declan G. Murphy, Damien Bolton, Nathan Papa

**Affiliations:** 1 Department of Surgery, Austin Health, University of Melbourne, Melbourne, VIC 3084, Australia; 2 Department of Surgical Oncology, Peter MacCallum Cancer Centre, Melbourne, VIC 3000, Australia; 3 Urology Service, Department of Surgery, Memorial Sloan Kettering Cancer Center, New York, NY 10065, USA; 4 Department of Urology, Western Health, Melbourne, VIC 3011, Australia; 5 School of Public Health and Preventive Medicine, Monash University, Melbourne, VIC 3004, Australia

**Keywords:** prostate cancer, TURP, radical prostatectomy, overall survival, population-based registry

## Abstract

**Background:** Following transurethral resection of the prostate (TURP) for lower urinary tract symptoms, incidental diagnosis of prostate cancer occurs in 2–10%. The significance of incidental prostate cancer on TURP compared to diagnosis via traditional diagnostic pathways is unclear. We aimed to compare post-prostatectomy outcomes in patients diagnosed with prostate cancer on TURP and prostate biopsy, using a population-based clinical quality registry. **Methods:** Data were extracted from the Victorian Prostate Cancer Outcomes Registry (PCOR-Vic). Patients who underwent prostatectomy between September 2008 and September 2020 and who were diagnosed by needle biopsy or TURP were included. The association between diagnosis method and overall survival was examined with Kaplan–Meier plots and the log-rank test. Multivariable Cox proportional hazards regression adjusting for PSA, age, year of surgery, diagnostic Gleason grade group and margin status was also used. Association with prostate cancer-specific mortality was examined with Fine and Gray competing hazards regression including the same covariates as above. **Results:** In total, 12022 patients met the inclusion criteria, of which 159 (1.3%) were diagnosed on TURP. After a median 58 months follow-up, diagnosis on TURP was associated with poorer 5-year overall survival at 91.2% (84.2–95.0%) compared to diagnosis on needle biopsy at 96.7% (96.2–97.0%), log-rank *p* = 0.001. Following multivariable adjustment, the hazard ratio of TURP vs. biopsy patients was 2.33 (95%CI: 1.35–4.01). For prostate cancer-specific mortality, there was a similar estimate; however, the 95% confidence interval crossed one, subHR = 2.24 (95% CI: 0.77–6.57). **Conclusions:** Diagnosis of prostate cancer on TURP was associated with poorer overall survival when compared to men diagnosed on prostate biopsy. These findings have clinical implications given the increased use of photoselective vaporisation of the prostate, which is characterised by the lack of pathologic tissue for assessment.

## Simple Summary

Sometimes prostate cancer is found by chance when men have surgery on the prostate to improve urinary symptoms (called TURP), rather than through a planned prostate biopsy. In this study, we looked at men who later had their prostate removed for cancer and compared outcomes between those whose cancer was first found on TURP and those diagnosed by biopsy. We found that men whose prostate cancer was discovered during TURP were more likely to die over time than men diagnosed by biopsy, even after taking into account age, PSA level, and cancer grade. Although prostate cancer-specific deaths were uncommon, the overall survival difference was clear. These results suggest that prostate cancer found incidentally during TURP may behave more aggressively or be harder to fully assess at the time of diagnosis. This is important for patients and doctors when deciding on follow-up and treatment after TURP, and as newer prostate procedures that do not provide tissue for testing become more common.

## 1. Introduction

Diagnosis of prostate cancer is typically established following prostate biopsy prompted by elevated serum prostate-specific antigen (PSA), abnormal digital rectal examination, and/or suspicious findings on multiparametric magnetic resonance imaging (mpMRI) [1]. Contemporary biopsy techniques, including both transrectal and transperineal approaches, are designed to provide systematic and targeted sampling of the peripheral zone of the prostate, which accounts for the majority of clinically significant prostate cancers [2]. In contrast, the transitional zone predominantly gives rise to benign prostatic hyperplasia (BPH), and malignant transformation in this region is less common and often biologically distinct.

Transurethral resection of the prostate (TURP) remains an effective and widely utilised surgical intervention for the management of bladder outlet obstruction secondary to BPH [3]. By virtue of its surgical approach, TURP selectively removes tissue from the transitional zone and does not aim to sample the peripheral zone [3]. As such, prostate cancer diagnosed following TURP is typically incidental, detected histologically rather than through targeted oncological investigation [4]. Reported rates of incidental prostate cancer following TURP range from approximately 2 to 10 percent [4,5,6].

Importantly, cancers detected incidentally on TURP may differ in their clinical presentation and diagnostic pathway compared with biopsy-detected disease, raising concerns regarding potential under-staging and delayed recognition of clinically significant tumours [4]. Whether these diagnostic differences translate into adverse long-term oncologic outcomes in patients proceeding to definitive treatment remains uncertain.

Management following the incidental diagnosis of prostate cancer on TURP is nuanced and depends on several factors, including patient age, life expectancy, comorbidities, pathological grade and volume of disease, and the likelihood of residual clinically significant cancer within the remaining prostate [5]. While many incidental cancers are low-risk and may be suitable for observation or active surveillance, a subset of patients are found to harbour intermediate- or high-risk disease. In these cases, further definitive treatment may be indicated to reduce the risk of disease progression and prostate cancer-specific mortality [1,7] Curative treatment options in this setting include radical prostatectomy or external beam radiotherapy, both of which may be technically and biologically influenced by the prior TURP procedure [8].

The oncologic implications of prostate cancer diagnosed incidentally on TURP, particularly among patients who subsequently undergo radical prostatectomy, remain incompletely defined. Historic series have suggested comparable oncologic outcomes between cancers diagnosed via TURP and those diagnosed by biopsy. However, these studies are limited by older diagnostic paradigms, heterogeneous staging, and a predominance of low-risk disease among TURP-detected cancers that proceeded to surgery [9]. Moreover, improvements in PSA screening, mpMRI utilisation, pathological grading, and surgical technique have substantially altered the contemporary prostate cancer landscape, limiting the applicability of older data to modern practice.

Accordingly, contemporary population-level data examining post-prostatectomy outcomes in patients diagnosed with prostate cancer via TURP compared with standard prostate biopsy are lacking. Using a population-based cancer registry, we sought to address this gap by comparing survival outcomes following radical prostatectomy between patients diagnosed with prostate cancer incidentally on TURP and those diagnosed via prostate biopsy. This analysis aims to clarify whether the route of diagnosis is independently associated with adverse oncologic outcomes and to inform counselling and management strategies for this unique patient subgroup.

## 2. Methods

Data for this study were obtained from the Victorian Prostate Cancer Outcomes Registry (PCOR-Vic), a population-based clinical quality registry that captures diagnostic, treatment, and outcome data for men diagnosed with prostate cancer across public and private institutions in Victoria, Australia. The registry methodology and governance framework have been previously described [10]. Vital status was determined through deterministic linkage with the Victorian Cancer Registry. Date of death was available up to 30 September 2020, while cause of death was available only for patients who died on or before 30 September 2019.

Men who underwent radical prostatectomy between September 2008, corresponding to the commencement of PCOR-Vic, and September 2020 were eligible for inclusion. Patients who underwent cystoprostatectomy were excluded to avoid confounding from concurrent bladder malignancy. To ensure contemporary and comparable treatment intent, only patients who proceeded to surgery within one year of prostate cancer diagnosis were included. Patients were excluded if vital status could not be ascertained, if prostate cancer was diagnosed by a method other than transrectal ultrasound-guided biopsy, transperineal biopsy, or transurethral resection of the prostate (TURP), or if key clinicopathological variables were missing. Specifically, cases with missing pre-treatment PSA, diagnostic grade group, or surgical margin status were excluded to allow robust multivariable adjustment. Patients with a recorded interval exceeding one year between diagnosis and surgery were also excluded to minimise lead-time and selection bias. Men were excluded if they received neoadjuvant therapy prior to radical prostatectomy, as such treatment is not the standard of care in this setting and would substantially confound oncologic outcome assessment.

Overall survival was defined as time from radical prostatectomy to death from any cause or last known follow-up. Survival outcomes were initially explored using Kaplan–Meier survival estimates, with differences between diagnosis groups assessed using the log-rank test. The association between route of diagnosis (TURP vs. biopsy) and overall survival was further examined using multivariable Cox proportional hazards regression. Covariates included age at surgery, time from diagnosis to surgery, year of surgery, pre-treatment PSA (log2-transformed), diagnostic International Society of Urological Pathology (ISUP) grade group, and surgical margin status. Proportional hazards assumptions were assessed using Schoenfeld residuals. Clinical and pathological T stage was not incorporated into the matching process, as tumour stage differed inherently by diagnostic pathway. Incidental cancers detected on TURP are classified as pT1a/b by definition, whereas biopsy-detected cancers are staged differently, making direct matching by T stage inappropriate and potentially misleading.

To further address potential confounding and baseline imbalance between groups, a matched cohort analysis was performed. One thousand independent 1:1 matched samples of patients diagnosed via TURP and biopsy were generated using calliper matching without replacement. Matching variables included PSA (within ±2.5 ng/mL), age (within ±2.5 years), and year of surgery (within ±1 year), with exact matching required for diagnostic grade group and surgical margin status. A pooled hazard ratio for overall survival was then derived across these matched samples.

Prostate cancer-specific mortality was analysed using Fine and Gray competing risks regression to account for death from non-prostate cancer causes as a competing event. The same covariates included in the overall survival models were incorporated into the competing risks analysis. Subdistribution hazard ratios with corresponding 95% confidence intervals were reported. Statistical significance was defined as a two-sided *p*-value < 0.05. All analyses were conducted using standard statistical software.

## 3. Results

A total of 13,386 open, laparoscopic, or robotic radical prostatectomies were recorded in the PCOR-Vic registry between September 2008 and September 2020 inclusive. After applying prespecified inclusion and exclusion criteria, 12,022 men (90 percent) were eligible for analysis (Supplementary Figure S1). Of these, 159 patients (1.3 percent) were diagnosed with prostate cancer incidentally following TURP, while the remaining patients were diagnosed via transrectal ultrasound-guided or transperineal prostate biopsy.

Baseline clinicopathological characteristics differed between the diagnostic groups. Men diagnosed via TURP had a lower median pre-treatment PSA compared with those diagnosed by biopsy (3.8 vs. 6.4 ng/mL) and a lower proportion of ISUP grade group greater than or equal to 2 disease (75 percent vs. 84 percent), consistent with the incidental nature of diagnosis in the TURP cohort (Table 1). Surgical approach and year of surgery were similar between groups.

**Figure 1:**
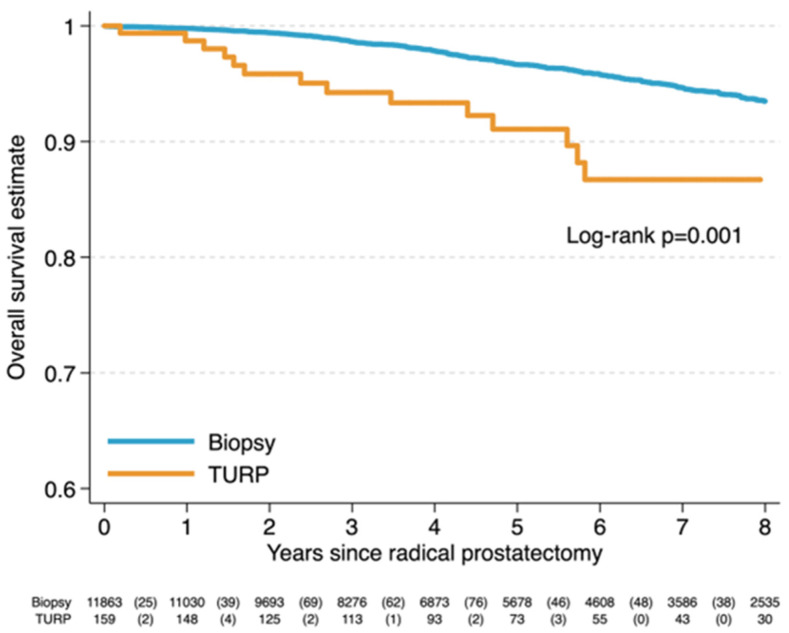
Kaplan–Meier estimates of overall survival by diagnosis method.

After a median follow-up of 58 months among surviving patients, 470 deaths from any cause were recorded, including 14 deaths in the TURP-diagnosed group. Kaplan–Meier analysis demonstrated a clear separation of overall survival curves between diagnostic pathways, with inferior survival observed among patients diagnosed via TURP (log-rank *p* = 0.001) (Figure 1). Estimated 5-year overall survival was 96.7 percent (95 percent confidence interval 96.2 to 97.0) in patients diagnosed by biopsy, compared with 91.2 percent (95 percent confidence interval 84.2 to 95.0) in those diagnosed following TURP.

On multivariable Cox proportional hazards analysis adjusting for age, time from diagnosis to surgery, year of surgery, log2-transformed PSA, diagnostic grade group, and surgical margin status, diagnosis via TURP remained independently associated with worse overall survival. The adjusted hazard ratio for death from any cause for TURP-compared with biopsy-diagnosed patients was 2.33 (95 percent confidence interval 1.35 to 4.01) (Supplementary Table S1). There was no evidence of violation of the proportional hazards assumption. Results were consistent in the matched cohort analysis, with a hazard ratio of 1.93 (95 percent confidence interval 1.11 to 4.77), supporting the robustness of the primary findings.

For prostate cancer-specific survival, follow-up was necessarily shorter due to the availability of cause-of-death data, resulting in a reduced analytic cohort of 11,204 patients. During this period, 351 deaths were recorded, of which 110 were attributed to prostate cancer, including four deaths in the TURP group. Competing risks regression demonstrated a similar magnitude of association between TURP diagnosis and prostate cancer-specific mortality compared with overall survival. However, due to the small number of prostate cancer-specific events in the TURP cohort, statistical precision was limited. The adjusted subdistribution hazard ratio was 2.24 (95 percent confidence interval 0.77 to 6.57), with the confidence interval crossing unity (Supplementary Table S2).

## 4. Discussion

Incidental prostate cancer diagnosed at the time of TURP for benign prostatic hyperplasia is uncommon, yet clinically important. In this large population-based cohort, men diagnosed with prostate cancer on TURP who subsequently underwent radical prostatectomy experienced significantly poorer overall survival compared with those diagnosed via prostate biopsy. This association persisted after multivariable adjustment and matched analyses, suggesting that the route of diagnosis itself may identify a subgroup of patients at higher risk of adverse outcomes. The mechanisms underlying this finding remain uncertain, but likely reflect a combination of disease heterogeneity, diagnostic limitations inherent to TURP, and the biological and surgical consequences of prior transurethral resection. These findings are particularly relevant in the contemporary era, where tissue-ablative procedures for BPH, such as photoselective vaporisation of the prostate (PVP), are increasingly utilised and do not yield tissue for pathological assessment.

The observation of inferior survival among TURP-diagnosed patients contrasts with earlier retrospective series that reported comparable oncologic outcomes between incidental and non-incidental prostate cancer diagnoses, including biochemical recurrence, prostate cancer-specific mortality, and overall survival [9,11]. However, these historic cohorts were dominated by men with Gleason 6 disease or lower, often comprising up to 90 percent of surgically treated patients. Such cohorts are not reflective of contemporary practice, where improved PSA screening, mpMRI utilisation, and risk stratification have shifted prostatectomy populations toward higher-risk disease. Given the low rates of biochemical recurrence and cancer-specific mortality associated with low-grade prostate cancer, the ability of earlier studies to detect meaningful differences in oncologic outcomes between diagnostic pathways was inherently limited. Notably, contemporary series evaluating post-prostatectomy outcomes specifically among men with incidental intermediate-risk or high-risk disease are largely absent from the literature.

Interestingly, a prior study by Nativ et al. [11] reported a signal toward poorer oncologic outcomes among men with higher-grade tumours diagnosed on TURP, with a markedly lower 5-year disease-free survival compared with biopsy-diagnosed counterparts (21 percent vs. 52 percent), although this analysis was limited by small numbers and lack of multivariable adjustment. Our findings build upon this observation in a modern, population-based cohort and suggest that the adverse outcomes associated with TURP-diagnosed disease may become more apparent as higher-risk tumours are increasingly represented among surgically treated patients.

Biological differences in tumour origin alone are unlikely to fully explain the observed survival disparity. Prostate cancers detected on TURP arise predominantly from transitional zone tissue. Earlier studies comparing transitional zone and peripheral zone index tumours reported broadly comparable post-prostatectomy outcomes, although these analyses were conducted more than a decade ago and involved predominantly low-risk disease [12,13,14,15]. More recent data by Teloken et al. demonstrated that transitional zone tumours were associated with higher rates of positive surgical margins, particularly at the bladder neck and apex, whereas peripheral zone tumours exhibited higher rates of other adverse pathological features, including extraprostatic extension, seminal vesicle invasion, and intraductal carcinoma [16]. In that study, peripheral zone tumours were associated with worse biochemical recurrence-free survival on both univariate and multivariable analysis.

The findings of the present study may differ from these prior reports due to the complex zonal architecture of prostate cancer. A proportion of patients diagnosed via TURP are likely to harbour concurrent transitional and peripheral zone tumours. Tumours involving multiple zones or exhibiting multifocal high-grade disease have been shown to behave more aggressively than solitary or single-zone tumours [17]. In this context, TURP may preferentially detect a component of disease while under-sampling clinically significant peripheral zone tumours, resulting in underestimation of disease burden at diagnosis.

An alternative and potentially complementary explanation relates to the impact of TURP itself on subsequent radical prostatectomy. Several studies have demonstrated that prior TURP is associated with increased technical difficulty at prostatectomy, particularly during dissection of the bladder neck and posterior prostate, and is linked to higher rates of positive surgical margins [18,19,20,21,22]. These effects are thought to arise from post-TURP inflammation, capsular disruption, fibrosis, and distortion of surgical planes [23]. While surgical margin status was adjusted for in the present analysis, margin positivity may represent only one downstream manifestation of broader operative and biological consequences.

Recent population-based data further support the concept that incidental prostate cancer detected on TURP may underestimate true oncologic risk. In a nationwide Danish registry study of men with Grade Group 1 and 2 prostate cancer diagnosed on TURP, long-term prostate cancer mortality was substantial, reaching 8.4% and 14% at 15 years, respectively, despite apparently low-grade disease at resection. The authors attributed this excess mortality to unsampled high-grade cancer in the peripheral zone. Notably, men with GG1 disease who underwent subsequent biopsy and had no residual cancer experienced negligible prostate cancer mortality, whereas those upgraded to GG2 or higher had markedly worse outcomes. These findings reinforce the need for systematic post-TURP evaluation to exclude occult clinically significant disease and align with the survival disadvantage observed in TURP-diagnosed patients undergoing radical prostatectomy in the present study.

With respect to oncologic outcomes, multiple groups have examined the risk of biochemical recurrence in men undergoing radical prostatectomy following TURP. Jin et al. reported that prior TURP was independently associated with an increased risk of biochemical recurrence after adjusting for age, PSA, and Gleason score (HR 2.27, 95 percent CI 1.13 to 3.94) [24]. Proposed mechanisms include microscopic dissemination of tumour cells during TURP, residual tumour burden in unsampled peripheral zone tissue, and alteration of the prostate cancer microenvironment following transurethral resection [24]. While these findings have been supported by other studies [25], conflicting results exist, with some series reporting no difference in biochemical recurrence-free survival [18,26,27]. Importantly, many of these studies were underpowered, were heterogeneous in design, and predated contemporary diagnostic and grading standards.

Although a numerically higher proportion of Grade Group 5 disease was observed in the post-TURP cohort compared with the biopsy-detected cohort (13% vs. 9%), this difference was modest and based on small absolute numbers, with wide and overlapping confidence intervals. The study was not powered to evaluate prognostic differences within this highest-risk subgroup, and it is therefore unlikely that this small imbalance alone explains the observed disparity in oncologic outcomes. Rather, the worse prognosis associated with incidental detection more likely reflects limitations inherent to TURP sampling, including incomplete assessment of residual prostate tissue and potential under-recognition of clinically significant peripheral zone disease.

The findings of this study have several important clinical implications for the contemporary management of men diagnosed with incidental prostate cancer following TURP. The observed association between TURP-detected prostate cancer and inferior oncologic outcomes highlights the limitations of assuming biological equivalence between incidental and biopsy-detected disease. These data support a low threshold for comprehensive post-TURP reassessment, including multiparametric MRI and confirmatory biopsy of unsampled prostate tissue, particularly in men with intermediate or higher-grade disease, adverse PSA kinetics, or longer life expectancy.

Second, these findings are increasingly relevant in the context of the expanding use of minimally invasive surgical therapies for benign prostatic hyperplasia, particularly tissue-ablative procedures such as photoselective vaporisation of the prostate (e.g., GreenLight PVP) [28,29,30]. Unlike TURP, purely ablative techniques do not yield histopathological specimens, thereby precluding incidental cancer detection and potentially allowing clinically significant prostate cancer to remain occult in a subset of men, even in the setting of low or declining PSA levels. The long-term incidence, natural history, and oncologic implications of such missed diagnoses remain poorly defined.

In contrast, enucleation-based approaches including holmium and thulium laser enucleation provide tissue for pathological evaluation through morcellation and allow incidental cancer detection. Accordingly, concerns regarding missed clinically significant prostate cancer primarily pertain to tissue-ablative modalities rather than enucleation techniques. As the adoption of these technologies continues to evolve, further prospective evaluation is required to define optimal cancer detection strategies, surveillance pathways, and outcomes across different surgical platforms.

Several limitations of this study warrant consideration. Detailed histopathological variables, including tumour focality, zonal origin, intraductal carcinoma, and cribriform architecture, were not available and may have provided additional biological insight into the observed outcome differences. Although extensive multivariable adjustment and propensity matching were performed, residual confounding cannot be excluded, particularly with respect to comorbidity burden, frailty, and functional status, which may influence overall survival independent of cancer-related factors. In addition, analyses of prostate cancer-specific mortality were limited by the relatively small number of cancer-related deaths in the TURP cohort.

As this study was based on a cancer registry capturing only patients diagnosed with prostate cancer, data on the total number of TURP procedures performed during the study period were not available. Consequently, the overall incidence of incidental prostate cancer following TURP and the proportion of all TURP patients diagnosed incidentally could not be determined. Nonetheless, the consistency of findings across multiple analytic approaches supports the robustness of the observed associations.

Exclusion of patients who received neoadjuvant therapy prior to prostatectomy may have further selected for patients treated according to standard clinical pathways. However, given that neoadjuvant treatment is not routine in contemporary practice and significantly alters pathological outcomes, inclusion of such patients would have introduced substantial confounding.

Finally, the exclusion of patients with an interval exceeding one year between diagnosis and radical prostatectomy, implemented to minimise lead-time and selection bias within the constraints of the registry structure, may have inadvertently favoured the inclusion of patients with more aggressive disease who proceeded to earlier definitive treatment. Conversely, patients with more indolent cancers who remained on active surveillance for prolonged periods, or who underwent delayed surgery beyond one year, were not captured in this analysis. This may have resulted in a study population enriched for higher-risk disease and should be considered when interpreting the generalisability of the findings to men managed conservatively.

## 5. Conclusions

In this large, contemporary population-based cohort, prostate cancer incidentally diagnosed following transurethral resection of the prostate was associated with significantly poorer overall survival after radical prostatectomy compared with cancer diagnosed via prostate biopsy. This association remained robust after comprehensive multivariable adjustment and matched analyses, indicating that the route of diagnosis identifies a subgroup of men at higher oncologic risk. The observed survival disadvantage is likely multifactorial, reflecting diagnostic under-sampling of clinically significant peripheral zone disease, tumour multifocality, and the biological and surgical consequences of prior transurethral resection. These findings underscore the importance of careful post-TURP evaluation, including consideration of mpMRI and confirmatory biopsy in selected patients, and highlight the need for caution as tissue-ablative procedures for benign prostatic hyperplasia become increasingly prevalent.

## 6. Take-Home Points

1. Prostate cancer incidentally diagnosed on TURP is uncommon but clinically meaningful, accounting for approximately 1–2 percent of men undergoing radical prostatectomy in this contemporary population-based cohort.
2. Men diagnosed via TURP had significantly worse overall survival after radical prostatectomy compared with those diagnosed by prostate biopsy, even after adjustment for age, PSA, tumour grade, year of surgery, and margin status.
3. The adverse survival signal persisted across multiple analytic approaches, including multivariable Cox regression and matched cohort analyses, suggesting that the route of diagnosis identifies a higher-risk subgroup rather than reflecting confounding alone.
4. These findings have important implications for post-TURP evaluation and BPH management, supporting thorough assessment of residual prostate tissue after incidental cancer detection and raising concern about tissue-ablative procedures such as photoselective vaporisation that preclude histopathological assessment.

## Figures and Tables

**Table 1:** Characteristics of the sample by diagnosis method, median (interquartile range) or *n* (%).

	TURP (*n* = 159)	Prostate Biopsy (*n* = 11,863)
Age at surgery (years)	66.9 (62.6–70.8)	64.6 (59.2–68.9)
Time of diagnosis to RP (days)	113 (74–199)	61 (41–98)
Year of surgery		
2008–2012	38 (24)	2967 (25)
2013–2016	66 (42)	4397 (37)
2017–2020	55 (35)	4499 (38)
Pre-surgery PSA (ng/mL)	3.8 (2.0–6.3)	6.4 (4.8–9.0)
Diagnostic grade group		
1	40 (25)	1895 (16)
2	67 (42)	5202 (44)
3	20 (13)	2483 (21)
4	12 (7.5)	1245 (10)
5	20 (13)	1038 (8.7)
Margin		
Negative	113 (71)	8798 (74)
Positive	46 (29)	3065 (26)

## Data Availability

Data are available on request due to restrictions (e.g., privacy, legal or ethical reasons).

## Supplementary Materials

The following supporting information can be downloaded at https://www.mdpi.com/article/10.3390/cancers18040569/s1: Figure S1: Study sample flow diagram; Table S1: Multivariable Cox regression to predict overall survival, n = 12,022 and 470 events; Table S2: Multivariable competing risks regression to predict prostate cancer-specific survival, n = 11,204, 110 events, 241 competing events.
