# Supplementary material for: Prostate Cancer Diagnosis by Transurethral Resection of the Prostate Is Associated with Compromised Oncologic Outcomes Post-Prostatectomy
Source: Cancers (Basel). 2026 Feb 9;18(4):569. doi: 10.3390/cancers18040569 (PMC12939765; doi:10.3390/cancers18040569)
Supplement: Supplementary file 1 [file cancers-18-00569-s001.zip › cancers-4120833-supplementary.pdf]

Supplementary Table S1. Multivariable Cox regression to predict overall survival, n=12022 and 470 events. HR = hazard ratio.

	HR (95% CI)
TURP vs Biopsy	2.33 (1.35 – 4.01)
Age at surgery, per five-year increase	1.21 (1.12 – 1.30)
Time diagnosis to RP, per 30-day increase	1.03 (0.98 – 1.08)
Year of surgery, per one-year increase	1.03 (0.98 – 1.08)
Log_2(PSA) (ng/ml)	1.12 (1.01 – 1.24)
Diagnosis grade group	
1	1.0
2	1.32 (0.96 – 1.83)
3	1.84 (1.30 – 2.60)
4	2.31 (1.59 – 3.35)
5	3.84 (2.67 – 6.54)
Margin	
Negative	1.0
Positive	1.81 (1.50 – 2.18)

Supplementary Table S2. Multivariable competing risks regression to predict prostate cancer specific survival, n=11204, 110 events, 241 competing events. SHR = sub-distribution hazard ratio. Grade group 1 and 2 combined due to few events in these individual categories.

	SHR (95% CI)
TURP vs Biopsy	2.24 (0.77 – 6.57)
Age at surgery, per five-year increase	0.94 (0.81 – 1.09)
Time diagnosis to RP, per 30-day increase	1.05 (0.93 – 1.19)
Year of surgery, per one-year increase	0.97 (0.90 – 1.04)
Log_2(PSA) (ng/ml)	0.95 (0.78 – 1.16)
Diagnosis grade group	
1/2	1.0
3	4.28 (2.14 – 8.57)
4	7.95 (3.92 – 16.1)
5	26.4 (13.9 – 50.0)
Margin	
Negative	1.0

Positive	3.52 (2.34 – 5.28)

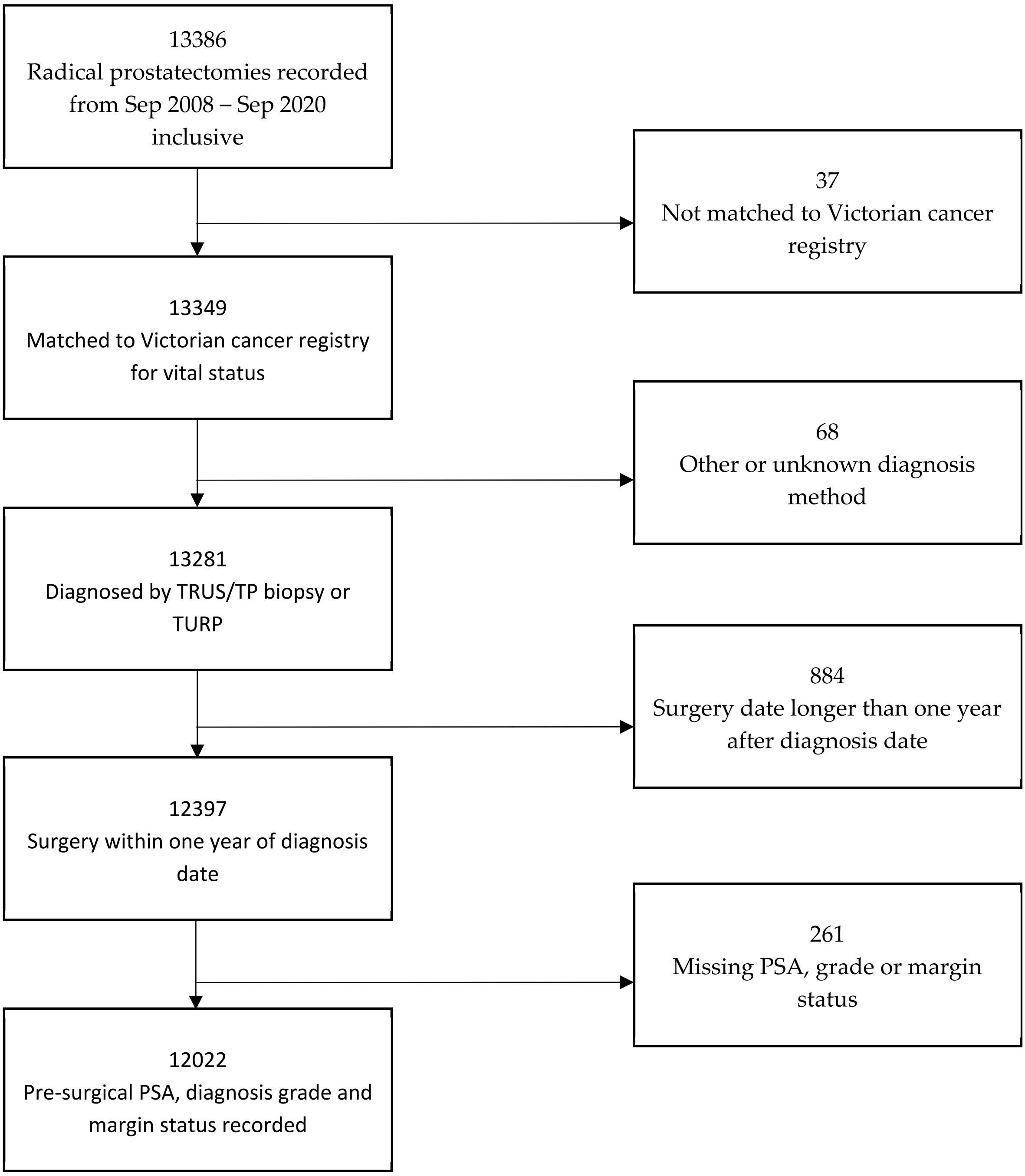

Supplementary Figure S1. Study sample flow diagram
